# Progression to Radiographic Axial Spondyloarthritis: A Narrative Review on Timeline and Novel Prediction Factors

**DOI:** 10.3390/ijms27114979

**Published:** 2026-05-30

**Authors:** Georgiana Eliza Murgu, Ioana Ruxandra Mihai, Ciprian Rezus, Maria Alexandra Burlui, Luana Andreea Macovei, Elena Rezus

**Affiliations:** 1 Department of Rheumatology and Rehabilitation, Grigore T. Popa University of Medicine and Pharmacy Iasi, 700115 Iasi, Romania; murgu.georgiana-eliza@d.umfiasi.ro (G.E.M.); maria-alexandra.burlui@umfiasi.ro (M.A.B.); luana.macovei@umfiasi.ro (L.A.M.); elena.rezus@umfiasi.ro (E.R.); 2 Department of Internal Medicine, Grigore T. Popa University of Medicine and Pharmacy Iasi, 16 University Street, 700115 Iasi, Romania; ciprian.rezus@umfiasi.ro; 3 IIIrd Internal Medicine Clinic, St. Spiridon County Emergency Clinical Hospital, 1 Independence Boulevard, 700111 Iasi, Romania; 4 Ist Rheumatology Clinic, Clinical Rehabilitation Hospital, 14 Pantelimon Halipa Street, 700661 Iasi, Romania

**Keywords:** axial spondyloarthritis, radiographic progression, structural damage, novel biomarkers

## Abstract

Spondyloarthritis represents a group of chronic immune-mediated rheumatic diseases that manifest as peripheral or axial musculoskeletal involvement. In axial spondyloarthritis (axSpA), the milestones of structural damage are represented by inflammation, followed by erosions in the sacroiliac joints (SIJ) and new bone formation. The purpose of this narrative review is to address the unmet needs regarding targeted risk stratification of disease progression in axSpA. While studies concerning predictive biomarkers have been conducted, their use in clinical practice has not yet been validated. Analysis of disease progression in patients recently diagnosed with non-radiographic axSpA, with fulfillment of the Assessment of Spondyloarthritis International Society criteria, determined a mean time of structural changes progression of 2.4 years. While factors such as human leukocyte antigen (HLA)-B27 and C-reactive protein are useful in classifying patients into risk categories regarding radiographic progression, novel biomarkers are needed in clinical practice to further facilitate treatment strategy selection. Choosing biomarkers to analyze the potential of both spinal and SIJ radiographic progression is useful in monitoring patients and reducing the burden of disease. Fetuin-A, sclerostin, and autoantibodies against Cluster of Differentiation 74 (anti-CD74) were associated with SIJ changes in various studies. Regarding spinal structural damage, adipokines, particularly leptin and visfatin, have been extensively studied and have shown promising results. Dickkopf-1, a regulator of the Wnt signaling pathway, vascular endothelial growth factor, and matrix metalloproteinase-3 have also presented associations with worsening modified Stoke Ankylosing Spondylitis Spine Score. The potential of each biomarker may be heightened by their use in prediction models with the purpose of implementation in clinical practice, particularly in improving patient outcomes and tailoring treatment strategies for individuals with spinal structural damage.

## 1. Introduction

Spondyloarthritis is a category of chronic immune-mediated rheumatic disorders characterized by peripheral or axial musculoskeletal involvement [1]. This classification comprises radiographic axial spondyloarthritis (r-axSpA), known as ankylosing spondylitis (AS); non-radiographic axial spondyloarthritis (nr-axSpA); reactive arthritis; psoriatic arthritis; enteropathic arthritis; and undifferentiated arthritis [2,3,4,5]. While the clinical presentation may be varied, this group of pathologies has many common features, such as genetic background [6], pathophysiology, disease pattern, and imaging findings [7,8].

Axial spondyloarthritis can be either radiographic (AS), which is defined by structural damage of the sacroiliac joints, or non-radiographic [9]. This latter form is outlined according to the Assessment of Spondyloarthritis International Society (ASAS) classification criteria [10,11]. From an imaging point of view, these criteria include magnetic resonance imaging (MRI) changes that are specific to sacroiliitis [12,13,14,15]. The structural changes are represented by widening or narrowing of the joint space, ankylosis, sclerosis, erosions, or fat lesions. Active lesions that show inflammation are defined by bone marrow edema (BME), enthesitis, capsulitis, and fluid in the joint space. The development of a standardized protocol for evaluation of the sacroiliac joints through the ASAS-SPARTAN collaboration facilitates the diagnosis of sacroiliitis [16].

The milestones of structural damage are represented by inflammation, followed by erosions in the sacroiliac joints (SIJ) and new bone formation. The precise order of each change is not yet known, but clinical studies suggest that these changes occur concomitantly. Fat metaplasia is thought to take place after the activation of the inflammatory process and before new bone formation [17,18]. In rare cases, fat metaplasia may be the only lesion, although it is most often associated with other changes, either structural or inflammatory [19].

The foundation of structural changes and promoter of specific damage in axSpA is determined by the trigger of inflammation. AxSpA is considered an autoinflammatory disease with an autoimmune component [20], with no current validated autoantibodies used in clinical practice for diagnosis [21,22,23,24]. Areas that are suggested to play a role in inflammation genesis are changes in the gut microbiome, genetic susceptibility, and biomechanical stress. The progression of the inflammatory process is a consequence of pro-inflammatory cytokines and an imbalance of the innate immune system, supported by various known risk factors. Human leukocyte antigen (HLA)-B27 is the most studied genetic factor involved in the pathogenesis of axSpA and has facilitated its diagnosis. Considering the pathogenetic status of axSpA, a disease that implicates both the autoinflammatory and autoimmune systems, there is the potential for identifying new markers for diagnosis, disease activity, and progression [25].

Taking into account factors involved in inflammation, new bone formation, and their potential role in structural damage, a targeted classification of biomarkers associated with progression can be developed to better define individual risk factors for each patient. In this narrative review, we emphasize the necessity of including predictive biomarkers to better characterize the pattern of progression in axSpA, and we integrate various factors that may be candidates for prediction models.

The literature search for this narrative review was conducted in electronic databases, PubMed, Google Scholar, and Web of Science. The search strategy included the following terms: “axial spondyloarthritis” AND (“progression” OR “progression factors” OR “radiographic” OR “radiographic progression” OR “biomarkers” OR “predictive biomarkers” OR “machine learning” OR “deep learning”). To ensure inclusion of all relevant studies, the timeframe for the literature search included publications from January 2004 to March 2026. Inclusion criteria consisted of articles published in English, human studies, clinical trials, meta-analyses, and articles published in peer-reviewed journals. Exclusion criteria comprised of abstracts, case reports, and non-peer-reviewed articles. The process of article selection was implemented through the analysis of titles, abstracts, and in-extenso articles.

## 2. Timeline of Structural Damage

Analysis of disease progression in patients recently diagnosed with non-radiographic axSpA, with fulfillment of the ASAS criteria, determined a mean time of structural changes’ progression of 2.4 years. A proportion of 16% of patients evolved to radiographic axSpA in this period. Factors associated with progression in this study were male sex, patients who achieved the imaging domain of the ASAS criteria, and favorable response to nonsteroidal anti-inflammatory drugs (NSAIDs) [26]. Another study, a five-year assessment of the prospective cohort DESIR (an acronym for “outcome of recent onset spondyloarthritis”), evaluated sacroiliac joint radiographic progression. Change from radiographic to non-radiographic sacroiliitis was established through the percentage of patients that achieved the New York modified criteria [27]. In total, 62 out of 416 patients (14.9%) fulfilled the classification criteria at baseline. At the five-year mark, this percentage increased to 20% in the population of patients that completed the study. In the “intention to follow” population, this proportion was 18% and 17.7% after imputation techniques, last observation carried forward, and linear extrapolation, respectively. Findings in this study underlined HLA-B27 positivity, increased C-reactive protein (CRP), and BME, as seen on MRI of the SIJ, as the main factors that predict risk of structural progression [28].

A more recent analysis of the DESIR cohort evaluated the proportion of progression over a 10-year follow-up. The population of patients that underwent imaging evaluation of the sacroiliac joints both at baseline and 10 years showed a net progression proportion of 5.8%. For patients from the “intention-to-follow” category that had at least one radiographic score from at least one reader, the shift to radiographic axSpA was estimated at 0.87 per year, with a progression of 8.7% at 10 years. In the case of time-varying therapy with tumor necrosis factor (TNF) antagonists, this estimation was reduced to 4.5%. Factors associated with fulfillment of the New York modified criteria were male sex, axial symptoms with a duration of >1.5 years, an Axial Spondyloarthritis Disease Activity Score (ASDAS) of ≥2.1 [29], smoking in HLA-B27 positive patients, and inflammation of the SIJ observed on MRI, particularly in cases with HLA-B27 positivity [30].

One study assessed the sensitivity to change in structural damage in patients from the DESIR cohort over a 10-year follow-up period. Imaging of the SIJ structural changes was conducted through radiographs and MRI at baseline and 1, 2, 5, and 10-year follow-ups. For statistical analysis of structural progression in the SIJ, fulfillment of the New York modified criteria was established as a binary variable for reference. The results showed the presence of at least three fatty lesions in the SIJ as the most sensitive change. The number of fatty lesions observed in a continuous field was the second most sensitive change. The detection of erosions was found to be the least sensitive outcome. This data supports the use of MRI over pelvic radiographs as the main tool of monitoring structural changes in axSpA [31].

A large, prospective, observational cohort also underlined the use of MRI in clinical trials to differentiate structural and inflammatory changes in the SIJ and to differentiate between radiographic and non-radiographic axSpA. As such, the study reported a limited use of the current differentiation between radiographic and non-radiographic disease regarding prediction of outcome and burden of disease. Patients with r-axSpA and grade 2 bilateral sacroiliitis had similar responses to treatment and progression of spinal radiographic changes [32], as assessed with the modified Stoke Ankylosing Spondylitis Spine Score (mSASSS) [33]. The study emphasized that patients with grade 2 bilateral sacroiliitis are similar in this regard to nr-axSpA, while patients with more severe SIJ changes have a lower probability of treatment effectiveness and a higher proportion of spinal radiographic damage. More severe SIJ damage was observed in male patients, whereas arthritis and enthesitis were corelated with less severe structural changes [32]. Although SIJ changes classify radiographic disease versus non-radiographic disease, spinal structural damage is essential when evaluating the patient concerning functional parameters, linking to disease activity and treatment response [34]. Chronologically evaluating spine radiographs using mSASSS may enhance the identification of structural deterioration, especially in monitoring disease development and its effects on patient outcomes [35].

## 3. Novel Biomarkers of Radiographic Progression

### 3.1. Regulators of the Wnt Pathway

The Wnt signaling pathway represents a key balancing element in new bone formation, one of the hallmarks of axial spondyloarthritis [36,37]. The main players of this regulatory process are Dickkopf-1 (DKK-1) [38] and sclerostin [39]. Sclerostin represents a protein secreted by mature osteocytes, which plays an essential role as an antagonist of the WNT signaling pathway [40]. The serum level of sclerostin has been negatively correlated with the presence of fatty lesions on MRI of patients with r-axSpA, as evaluated with the Berlin scoring method. This suggests a role of sclerostin levels in structural changes’ progression. Treatment with TNF antagonists determined a significant increase in sclerostin concentration [41]. Dickkopf-1, a member of the DKK family, is a protein expressed by osteoblasts, osteocytes, and various organs. DKK-1 is also an antagonist of the canonical signaling Wnt, with the ability to regulate the Wnt pathway [42]. Evaluation of DKK-1 and serum sclerostin levels in the DESIR cohort found a higher level of DKK-1 in patients with axSpA compared to controls. As a marker of bone resorption, DKK-1 may be linked to erosive lesions, but results were inconclusive, given the cohort characteristics, such as the variability in disease severity and treatment history among patients. In the same study, serum sclerostin was found to be decreased compared to controls. There was a significant association in the studied population between serum sclerostin values and SIJ changes on radiography [43]. In a study that compared DKK-1 and sclerostin levels in r-axSpA, rheumatoid arthritis (RA), and healthy controls, both proteins were found to be increased in patients with r-axSpA compared to the other groups. While sclerostin values presented a negative association with mSASSS, DKK-1 had a positive correlation, but without statistical significance [44]. Although values of these proteins vary across different cohorts, most studies observed lower serum levels of sclerostin in axSpA [41,43,44,45,46,47,48], an association with structural changes, and an increase [43,46] in values after TNF inhibitor treatment [41,45,47,48].

### 3.2. Adipokines

Adipokines represent bioactive factors secreted by adipocytes, with roles in metabolism and the homeostasis of inflammation. Adiponectin and leptin are also key elements in immune response [49]. Adiponectin influences the immune system through its different isoforms. The high-molecular-weight isoform has a protective cardiovascular effect, while effects in rheumatic inflammatory diseases are contradictory, with some studies suggesting a potential exacerbation of inflammation in these conditions [50]. Leptin interacts with both the innate and the adaptive immune systems and determines the increase in pro-inflammatory cytokines [50]. Clinical studies have had varied results concerning serum levels of leptin and adiponectin in patients with axial spondyloarthritis compared with controls [51,52,53,54]. One study underlined an association between higher levels of leptin in patients with r-axSpA and the presence of syndesmophytes on spinal radiography but not disease activity. Serum leptin concentration was higher in these patients compared to those with r-axSpA and a lack of syndesmophytes and healthy controls. There was no difference in adiponectin levels in the three groups of patients, and a connection was observed between serum adiponectin and radiographic changes [54]. In a study that included 137 patients with r-axSpA that had not yet started treatment with TNF inhibitors, adipokine levels were measured at baseline and after starting biologic treatment. The high-molecular-weight isoform of adiponectin remained stable during treatment. Baseline concentration of leptin was correlated with radiographic progression after 4 years. Furthermore, changes in serum leptin levels within 2 years of treatment had a statistically significant association with mSASSS progression after 4 years [55]. In a study that included results from the ENRADAS trial, adipokine levels were investigated in relation to radiographic structural changes’ progression. The patients that took part in this study were all diagnosed with r-axSpA and had a symptom duration of 14.8 ± 12.2 years. Patients were treated with NSAIDs only, continuously or on demand. Male patients had lower concentrations of serum leptin and high-molecular-weight adiponectin compared to female patients. Analysis showed an association between lower values of these adipokines and radiographic progression of the spine only in male patients. The study showed that increased levels of leptin and the high-molecular-weight isoform of adiponectin predicted a protective effect against radiographic spinal changes in r-axSpA patients. According to this study, higher levels of these molecules in female patients may explain the lower prevalence of new bone formation in the spine in women diagnosed with axSpA [56].

Visfatin, also known as pre-B cell colony-enhancing factor, is predominantly produced in the visceral adipose tissue. It is an adipokine that demonstrates important metabolic roles and mediates the innate immune system in chronic inflammation of the synovial membrane. Results of clinical studies suggest an association with disease activity in RA [57]. Syrbe et al. found that baseline levels of visfatin were found to be significantly higher in patients with r-axSpA than in healthy controls. The study found no association between factors such as age, disease duration, treatment with disease-modifying antirheumatic medication (DMARD), CRP, and serum concentration of visfatin. In terms of subsequent structural damage progression, baseline visfatin levels were corelated with the formation/progression of syndesmophytes and mSASSS progression. Serum visfatin with a cut-off value of 8 ng/mL was equivalent to a CRP concentration of 6 mg/liter in terms of radiographic damage progression prediction in r-axSpA [58]. Rademacher et al. observed that baseline visfatin was associated with radiographic progression at two years. Change in visfatin serum concentration after two years was associated with spinal structural damage progression at 4 years [55]. Another study found a positive association between levels of serum visfatin and mSASSS. The same study found a positive correlation between visfatin concentration and Bath Ankylosing Spondylitis Disease Activity Index (BASDAI) in nr-axSpA patients [59] but not in the r-axSpA group [60]. Increased levels of serum visfatin were also linked to the presence of fat deposition in the lumbar spine on MRI in r-axSpA patients, with a potential role in the pathophysiology of new bone formation and maybe an independent risk factor for lumbar spine fat deposition in r-axSpA patients [61]. Resistin is an adipokine with high metabolic impact, connected to insulin resistance. Studies have shown that resistin can induce the production of interleukin-6 (IL-6), IL-1, and TNF-alpha [62]. In a preliminary longitudinal prospective study on 20 patients with r-axSpA, resistin levels were significantly higher in axSpA patients than in healthy controls. High concentrations of resistin at baseline showed correlations with progression of mSASSS over two years [63]. Syrbe et al. also found higher levels of resistin at baseline compared to controls. In this study, female patients with axSpA were found to have higher concentrations of serum resistin than male patients. Resistin levels correlated with CRP concentration, but not with radiographic progression [58]. Analysis of adipokines in further research as potential biomarkers of radiographic progression in axSpA patients is supported by the statistically significant results obtained in these studies (Table 1).

### 3.3. Autoantibodies Against Cluster of Differentiation 74

Cluster of differentiation (CD74), also known as the HLA class II histocompatibility antigen gamma chain, consists of two elements: thyroglobulin type-1 and the class II-associated invariant chain peptide (CLIP). CD74 plays a role in the formation of the major histocompatibility complex class II (MHCII) and influences differentiation of B cells. Anti-CD74 IgG autoantibodies were identified in 67% of patients with spondyloarthritis (SpA) by implementing the ELISA technique on years-long frozen sera. In patients with axial symptomatology of less than a year, anti-CLIP autoantibodies were found in a proportion of 97% in patients with r-axSpA and peripheral SpA. The percentage of patients with anti-CD74 antibodies declined after one year of disease duration [64]. Baraliakos et al. also found that anti-CLIP antibodies have shown a strong association with r-axSpA, with a sensitivity for diagnosis of 85.1% and a specificity of 92.2% [65]. Ziade et al. found no difference in levels of IgG anti-CD74 autoantibodies between non-radiographic and radiographic axSpA [66]. Another study found no connection between anti-CD74 autoantibodies and the age of the patients. There was no difference between early and late axSpA regarding frequency of autoantibodies. The proportion of anti-CD74 IgG antibodies was higher in patients with increased disease activity [67]. Lan Do et al. have analyzed the link between anti-CD74 IgA autoantibodies and radiographic progression in r-axSpA. The study found a significant association with the presence of syndesmophytes and severe radiographic changes. There was no identified predictive value of anti-CD74 IgA autoantibodies and structural changes of the spine [68]. Hu et al. found no predictive value of anti-CD74 autoantibodies for the diagnosis of axSpA or disease manifestations. The study included 97 patients with axSpA, both radiographic and non-radiographic forms. Anti-CD74 IgA and IgG antibody serum levels were evaluated in 21 patients before and after therapeutic intervention. While IgG antibody concentrations were reduced after treatment, no statistically significant changes were observed in IgA levels. The study confirmed a positive association between anti-CD74 IgA antibodies, HLA-B27 status, and BASDAI in these patients. No diagnostic value was demonstrated for either IgA or IgG antibodies and predictive utility was not proven in the Chinese axSpA patients. This shows a possible racial or geographical trait [69]. Research assessing data from an observational cohort and the ENRADAS cohort examined the possible association between anti-CD74 autoantibodies and sacroiliac joint and spinal alterations in individuals with axial spondyloarthritis. Anti-CD74 IgA antibodies were significantly correlated with grade 4 sacroiliitis in the observational cohort. In the ENRADAS cohort, the presence of these autoantibodies was associated with higher mSASSS at baseline and mSASSS increase after 2 years. Results underlined anti-CD74 IgA antibodies as a potential predictive biomarker for structural damage progression in axSpA [70]. Correlations between anti-CD74 antibodies and structural changes suggest utility as potential predictive biomarkers in certain populations and the need for further studies and validation (Table 2). Some unmet needs are represented by geographical stratification of anti-CD74 antibodies in axSpA patients, analysis of disease severity, and patient profile, especially regarding risk of radiographic progression, and direct comparison with HLA-B27 in these aspects.

### 3.4. Fetuin-A

Fetuin-A is a plasma carrier protein that plays an essential role in immune response. It has both anti-inflammatory and pro-inflammatory effects depending on the activating factor. Fetuin-A has been demonstrated to display anti-inflammatory characteristics and may play a role against diseases such as rheumatoid arthritis [71]. Levels of fetuin-A have been shown to be decreased in rheumatic diseases compared to healthy controls [72,73]. A research revealed elevated blood fetuin-A levels in r-axSpA patients relative to healthy controls, independent of age, sex, treatment, disease activity, and metabolic syndrome-related characteristics [74]. This result was in agreement with findings from Harman et al. that found higher concentrations of fetuin-A in axSpA and RA patients. Patients with peripheral SpA had similar levels of fetuin-A as healthy subjects. In the axSpA group, serum levels were correlated with disease progression, suggesting a potential role as a marker for new bone formation [75]. A case–control study found an association between fetuin-A serum levels and syndesmophytes. The research found that patients with syndesmophytes had higher levels of fetuin-A than those without these structural changes. There was no difference between the latter group and healthy controls regarding fetuin-A concentration [76]. Results from an analysis of the SpondyloArthritis Caught Early (SPACE) study cohort found lower levels of fetuin-A in patients with SIJ structural changes. This correlation was established for patients with early axSpA and was found to persist after a period of two years. There was no association between low concentrations of fetuin-A levels and spinal structural damage. These results underlined the potential of the baseline of fetuin-A as a biomarker for SIJ radiographic progression [77].

### 3.5. Matrix Metalloproteinase-3

Although matrix metalloproteinase-3 (MMP-3) has been mostly associated with disease activity in axSpA [78,79,80,81], clinical studies have shown a potential correlation with structural progression. MMP-3 has been underlined as an independent prediction factor for radiographic damage of the spine in r-axSpA patients. This association was mainly observed in patients with preexisting structural changes at the time of evaluation. High levels of baseline MMP-3 and baseline mSASSS with a cut-off value of 10 units were correlated with a significant risk of progression of structural damage [82]. In the Italian arm of the SPACE study, MMP-3 was shown to be significantly correlated with mSASSS [81,83], but not with SIJ changes [83].

### 3.6. Serum Calprotectin

Calprotectin is a calcium-binding protein, part of the S-100 protein family, which has antimicrobial characteristics and frequently correlates with ESR and CRP levels in acute-phase inflammatory reactions [84]. Calprotectin may play a pathophysiological role in axSpA [85], although certain studies have not found an association between serum levels and BASDAI, BASFI [86], and BASMI [85,87]. Baseline serum calprotectin was shown to be correlated with mSASSS worsening [55,88], new syndesmophyte formation after 2 years [55], and, consequently, radiographic progression in axSpA, although not all studies have found a significant association with structural damage [89]. Data from the DESIR cohort has shown a link between serum calprotectin, inflammatory parameters, and MRI inflammatory changes of the spine and SIJ in early axSpA, but not structural damage [90]. Serum calprotectin has also been associated with severity of disease [91] and ASDAS [92,93].

### 3.7. Vascular Endothelial Growth Factor

Vascular endothelial growth factor (VEGF) is a key regulator of angiogenesis, playing a role in endochondral ossification and new bone production [94]. VEGF levels were found to be considerably greater in r-axSpA patients with high disease activity and restricted function than in healthy controls. VEGF levels were similarly linked to smoking exposure in these individuals [95]. VEGF was found to be a promising marker of disease activity, with good correlation with BASDAI [96]. Poddubnyy et al. have shown that high levels of baseline VEGF represent an independent risk factor for spinal radiographic progression. In patients who presented with syndesmophytes at baseline, the sensitivity and specificity of the predictive value of VEGF above levels of 600 pg/mL were 47–53% and 94–97%, respectively [97]. VEGF levels were also associated with BASFI, mSASSS, and ASDAS [98].

### 3.8. Alkaline Phosphatase

Alkaline phosphatase (ALP) represents a known marker of bone turnover, particularly bone-specific ALP [99]. A retrospective study analysed data from 1280 patients with ankylosing spondylitis from diagnosis. Further analysis included 1122 patients that had at least two assessments of serum ALP and mSASSS, at an interval of more than 90 days. The aim of this study was to evaluate the correlation between serum ALP levels and radiographic progression of the spine. The follow-up period had a mean value of 8.2 years. The study found that ALP levels 5 years prior to radiographic progression may be a potential predictive factor for structural changes of the spine in axSpA. Results of this study underline the need for prospective studies with a follow-up period of more than 5 years to establish biomarkers of radiographic progression [100]. In another study, Liu et al. obtained mesenchymal stem cells (MSCs) from the enthesis of the spine pertaining to patients with ankylosing spondylitis. These cells exhibited accelerated mineralization, regulated by tissue-nonspecific alkaline phosphatase (TNAP) [101]. This enzyme plays an essential molecular function in bone mineralization [102]. High levels of expression of TNAP and increased activity of ALP were strongly connected to increased mineralization in MSCs in patients with axial spondyloarthritis compared with controls. Unfolded HLA-B27 determined upregulation of TNAP in MSCs, acting as a catalyst for syndesmophyte formation. In mice models, inhibition of TNAP blocked the formation of bony appositions after implantation of MSCs from the enthesis involved in spinal ankylosis. Samples from two cohorts of patients with radiographic axial spondyloarthritis were assessed to detect levels of bone-specific TNAP (BAP). Statistical analysis revealed that disease duration, serum BAP levels, and/or CRP levels were independent factors of prediction for severity of structural changes in axial spondyloarthritis. Further longitudinal analysis showed that CRP concentration and serum BAP levels were predictive factors for yearly radiographic changes [101].

## 4. Artificial Intelligence

Artificial intelligence may prove useful in the complex challenges of diagnosing axSpA. This technology can complement clinical judgment and imaging investigations in day-to-day practice, particularly by providing additional insights and data analysis that can enhance decision making for patient care. Deep learning and machine learning may have a role in monitoring and predicting radiographic progression in axSpA [103]. In a retrospective study that analyzed data from 412 patients with axSpA according to the ASAS, seven machine learning models were evaluated to investigate the potential of radiographic progression prediction. Multivariable logistic regression analysis revealed the presence of syndesmophytes at baseline evaluation, body mass index, male gender, and high values of ESR as independent prediction factors. Baseline mSASSS did not predict structural damage in this analysis. The prediction models demonstrated mSASSS was the most important variable in most algorithms. The presence of syndesmophytes at baseline and sacroiliitis grades was also useful to predict radiographic progression. Balanced accuracy in all models was approximately 65%, which is less than satisfactory. The main cause for this low predictive value is represented by the small sample size, not sufficient for optimal training. The study underlined the necessity for more data regarding new bone formation, such as MRI analysis and molecular profiles. Patient heterogeneity was also a main factor for reduced predictive ability of the models, as both non-radiographic and radiographic axSpA patients were included. This translates to mixed baseline spinal changes profiles [104]. This study underlines limitations in establishing machine learning models in prediction medicine: the need for large sample sizes, complex data for each patients profile, including clinical, imaging, and molecular profiles, and a relatively homogenous population for each criterion, in particular imaging techniques and characterization. Baek et al. evaluated the potential of artificial neural networks (ANNs) in the prediction of spinal structural damage progression based on mSASSS. ANN displayed a better ability of prediction compared to the generalized linear model. The most important variables for the ANN model were mSASSS segment scores with values of 3 and 2, followed by scores of 0 and 1. Other important factors were time after the first evaluation, history of uveitis, smoking, and TNF antagonist treatment period [105]. A multicentric retrospective study evaluated the role of deep learning technology centered on anatomy in predicting sacroiliitis based on SIJ radiographs. The cohort included data from four studies: Multicountry Registry of Clinical Characteristics (PROOF) [106]; German Spondyloarthritis Inception Cohort (GESPIC) [107]; Identification of the Optimal Referral Strategy for Early Diagnosis of Axial Spondyloarthritis (OptiRef) [108]; and Diagnostic Accuracy of MRI and CT in axSpA (DAMACT) [109,110]. SIJ radiographs were channeled through an anatomy-based pipeline to interpret the results through deep learning. The results have shown that incorporating anatomy into artificial intelligence models can improve ability in predicting definite sacroiliitis. This model displayed the capacity to identify high-risk patients, detecting subtle radiographic changes in cases not yet established as definite sacroiliitis, thus defining a trajectory of structural damage progression [111]. A recent retrospective cohort study sought to determine factors associated with both risk and protection of radiographic progression using a deep learning model. Imaging evaluation was conducted by lateral radiographs of the cervical and lumbar spines, and changes were scored with mSASSS. Age at baseline evaluation of mSASSS, predominantly in patients older than 40 years, was shown to be predictive of disease progression. Baseline mSASSS > 5 was also associated with further spinal changes. Continuous full-dosage exposure to NSAIDs was associated with reduced radiographic progression [112]. Garofoli et al. directly analyzed traditional prediction models and machine learning models in a prospective DESIR cohort study. The research compared these methods in patients with early axial spondyloarthritis. Radiographic progression was evaluated in both the spine and the SIJ joints. Results were similar in machine learning and traditional models and the study failed to confirm its hypothesis of machine learning superiority, with a best cross-validated area under the curve (AUC) of 0.79 for traditional models and 0.77 from machine learning models. As stated in the study, one of the disadvantages of machine learning is the high levels of data necessary to achieve proper results compared to logistic regression, reaching as much as 10 times more events for each variable [113].

While prediction models can prove their usefulness in the prediction of radiographic progression, it is of paramount importance to assess biomarkers regarding their potential to predict changes in the SIJ in order to be better implemented in clinical practice (Table 3).

A recent study aimed to identify molecular biomarkers associated with radiographic progression and severity of disease. The study included 144 patients with axSpA composed of three cohorts (exploratory cohort, validation cohort, and severity cohort). Collected data included demographic factors, disease activity scores, functional assessment, mSASSS, and inflammatory markers (ESR, CRP). The first cohort comprised 24 patients with axSpA, in which samples were utilized for RNA sequencing from peripheral blood mononuclear cells. This group of patients had a 5-year follow-up for monitoring radiographic progression. The second cohort, which included 60 patients, was used to validate data from the exploratory cohort. Patients underwent proteomic profiling using 92 inflammatory proteins. A separate cohort was analyzed to establish the involvement of inflammatory markers in disease severity, including the inflammatory proteome. Boruta, a machine learning method, was used in the first cohort to identify genes that could play a role as prediction biomarkers for radiographic changes in axSpA patients. The most important molecular factors of prediction included EIF3A, EMP3, S100A4, KAT2B, TMF1, and FBXW2. Patients were classified as slow, moderate, and fast progressors regarding radiographic changes. In the severity cohort, moderate and fast progressors had elevations in the levels of 13 proteins (IL10, IL10RA, IL10RB, IL15RA, IL2, IL2RB, IL12B, IL24, SLAMF1, NT3, PDL1, CD6, CCL19) [114].

## 5. Discussions

Perspective concerning axSpA has evolved considerably in the last decade, from nomenclature to treatment options and monitoring. While treatment is selected according to patient profile, including extramusculoskeletal manifestations [115], personalised therapy has not yet reached its full potential. In order to achieve this and reduce the burden of disease, early diagnosis and effective monitoring should be accomplished. Imaging techniques, such as MRI, play an important role in these aspects, and current protocols are in a constant process of adjustment [14,15,16]. Although the role of MRI is well established, interpretation of images of the SIJ have not reached the desired level of specificity in clinical practice. BME is not a highly specific change, as it can be encountered in healthy populations, post-partum women, and athletes [116].

In a recent study by de Bruin et al., data from the Spondyloarthritis Caught Early (SPACE) cohort was used to evaluate predictive validity of active and structural lesion definitions of the SIJ as seen on MRI. The study compared the existing ASAS-MRI-SIJ+ definition with the definition proposed by the MRI study group and evaluated the utility of structural lesions when combined with active lesions for diagnosis. The Leiden definition for structural changes was validated, as opposed to the definition proposed by the MRI study group. The definitions from both groups for active lesions were validated. Structural changes, as established by the two definitions, increased sensitivity for axSpA diagnosis by only 6–11%. Structural changes were found to have limited utility in early diagnosis of axSpA [117]. Rates of structural progression, particularily in the context of New York criteria, are also essential. The available studies underline a proportion of progression of 10–40% within a timeframe of 2–10 years. Inflammatory changes observed on SIJ MRI, high CRP, positive HLA-B27, and a history of smoking are factors correlated with progression of disease from non-radiographic to radiographic axSpA [118]. High disease activity according to ASDAS was associated with radiographic spinal progression in early axSpA. Comparatively, mSASSS progression of at least 2 points was observed in 20% of patients with radiographic axSpA, as opposed to 9% of patients with non-radiographic axSpA over a 2-year period [119]. A proper timeline of structural change progression depending on patient profile is still a challenge, as male gender, obesity, smoking, CRP, ESR levels, ASDAS, BASDAI, BME, and fatty lesions on MRI are all associated with spinal radiographic progression [120]. Selection of predictive biomarkers should take into account these factors to ensure value as an independent variable. Other elements that need consideration are availability for use in clinical practice, cost, maintaining a high level of performance across different gender and racial populations, and validation of cut-off values, especially when considering prediction models. Given that, in terms of radiographic progression, most studies found that SIJ changes precede spinal changes, biomarkers associated with SIJ structural progression could be a starting point to include in further research. Although results vary across clinical research, sclerostin, fetuin-A, and, potentially, anti-CD74 antibodies could show promise in this endeavor. One of the main issues regarding validation of biomarkers for clinical practice is the heterogenous population included in the analyzed studies. In order to properly compare different molecules for their predictive potential, a similar patient population should be included. This implies disease duration, HLA-B27 status, axSpA subtype, radiographic or non-radiographic, and exposure to treatment, including NSAID. The mechanism of specific therapy should also be considered, be it TNF antagonists, IL-17 inhibitors, or Janus kinase inhibitors, as well as duration of exposure. Baseline radiographic changes of the spine and SIJ are essential, including mSASSS score and cut-off, and sacroiliitis stage. Variability of patient cohort and the multiple factors that can influence radiographic progression represent the main challenges in validating prediction biomarkers for use in clinical practice.

## 6. Conclusions

Current international guidelines have highlighted the necessity of preventing disease progression in axSpA patients. While standard factors such as CRP and HLA-B27 have proven useful in discerning patients at high risk for structural damage, the plethora of biomarkers identified in clinical studies allows a possible selection for use in clinical practice. Although studies that analyze prediction factors for SIJ damage progression are relatively scarce in comparison, biomarkers for spinal structural changes have been more extensively studied. There are various candidates for prediction markers of radiographic progression in axSpA, many of them also associated with disease activity. Anti-CD74 autoantibodies have been associated with both sacroiliitis and spinal changes, while their predictive value needs to be validated in further studies. Sclerostin and fetuin-A have shown promise as prognostic biomarkers for SIJ alterations.

## 7. Future Perspectives

Classifying biomarkers in spinal or SIJ radiographic progression factors may have a high utility in personalizing the risk profile of patients with axSpA and targeting monitorization of disease progression. Further studies are needed to improve selection of biomarkers for prediction models. Beyond inflammation markers and HLA-B27, analysis of baseline radiographic changes and baseline biomarkers for structural damage prediction should be implemented. The cumulation of prediction factors and their use in prediction models can facilitate risk stratification and targeted selection of therapy. Future research should aim to validate biomarkers for early diagnosis and defining patient phenotype, especially concerning the prospect of severe disease activity and rapid structural progression.

## Figures and Tables

**Table 1:** Adipokines in axial spondyloarthritis radiographic progression.

Authors	Year	Studied Adipokines	Type of Structural Change	Results
Gonzalez-Lopez et al. [54]	2017	Leptin Adiponectin	Syndesmophytes	Higher levels of serum leptin were observed in patients with syndesmophytes compared with those without syndesmophytes. No association was made between adiponectin concentration and the presence of syndesmophytes.
Rademacher et al. [55]	2022	Adiponectine (HMW isoform), Leptin, Visfatin	Syndesmophytes, mSASSS progression	Baseline visfatin levels had an association with mSASSS progression of ≥2 points and were correlated with mSASSS worsening of ≥4 points after 4 years. Changes in concentration levels of leptin and visfatin after 2 years were associated with mSASSS progression after 4 years. No statistically significant association was made between adiponectin levels and radiographic progression.
Hartl et al. [56]	2017	HMW adiponectin, Leptin, Visfatin, Resistin, Chemerin, Lipocalin-2, Omentin	mSASSS progression, Syndesmophytes	Serum concentrations of HMW adiponectin and leptin showed a significant inverse association with radiographic progression in male patients with r-axSpA. Higher levels of the adipokines in female patients suggest a protective role against structural damage progression.
Syrbe et al. [58]	2015	Adiponectin, Visfatin, Resistin	mSASSS progression, Syndesmophytes	Baseline levels of visfatin were associated with mSASSS change by ≥2 points after 2 years and syndesmophytes formation or progression. Adiponectin and resistin levels were not significantly correlated with mSASSS changes, syndesmophyte formation, or progression.
Hulejová et al. [60]	2019	Visfatin	mSASSS change	Visfatin serum levels were shown to be higher in patients with mSASSS ≥ 1.
Shen et al. [61]	2024	Visfatin	Fat deposition in the lumbar spine	High levels of serum visfatin were shown to be an independent risk factor for fat deposition in the lumbar spine.
Park et al. [63]	2017	Adiponectin, Leptin, Resistin	mSASSS progression	Differences in levels of leptin and leptin/BMI after 2 years were correlated with mSASSS progression. Baseline levels of serum resistin were associated with mSASSS changes.

Abbreviations: HMW = high molecular weight; mSASSS = modified Stoke Ankylosing Spondylitis Spine Score; r-axSpA = radiographic axial spondyloarthritis; BMI = body mass index.

**Table 2:** Association between anti-CD74 antibodies and radiographic progression in axial spondyloarthritis.

Authors	Year	Type of Immunoglobulin	Type of Structural Change	Results
Ziade et al. [66]	2019	IgG	Sacroiliac joint changes	No difference in anti-CD74 IgG4 was observed between patients with radiographic and non-radiographic axSpA.
Do et al. [68]	2021	IgA	mSASSS changes Syndesmophytes	Log anti-CD74 IgA has a significant association with mSASSS changes and syndesmophyte presence.
Witte et al. [70]	2020	IgA	mSASSS Grade of radiographic sacroiliitis	Anti-CD74 IgA autoantibodies were associated with a higher baseline mSASSS and a higher mSASSS progression in the following 2 years. Anti-CD74 IgA antibodies had a statistically significant correlation with grade 4 sacroiliitis (52.9% in IgA-positive patients). versus 25% in IgA negative patients).

Abbreviations: Ig = immunoglobulin; CD = cluster of differentiation; axSpA = axial spondyloarthritis; mSASSS = modified Stoke Ankylosing Spondylitis Spine Score.

**Table 3:** Molecules associated with radiographic progression of the sacroiliac joints.

Authors	Year	Biomarker	Results
Nocturne et al. [43]	2015	Sclerostin	Low levels of serum sclerostin were associated with radiographic sacroiliitis (*p* = 0.023).
Favero et al. [77]	2023	Fetuin-A	Low fetuin-A levels at baseline were shown to be an independent risk factor for radiographic sacroiliitis (*p* = 0.048).
Witte et al. [70]	2020	Anti-CD74 IgA antibodies	Anti-CD74 IgA was significantly associated with grade 4 sacroiliitis (*p* = 0.004). Patients with anti-CD74 IgA antibodies were shown to have a higher mean sacroiliitis grade (*p* = 0.01).

Abbreviations: CD = cluster of differentiation; Ig = immunoglobulin.

## Data Availability

No new data were created or analyzed in this study. Data sharing is not applicable.
